# Supplementary material for: Are carbon emissions trading and green financial instruments synergistic? -Comprehensive quantitative research based on content analysis
Source: PLoS One. 2024 Mar 7;19(3):e0298601. doi: 10.1371/journal.pone.0298601 (PMC10919601; doi:10.1371/journal.pone.0298601)
Supplement: S1 Data — (ZIP) [file pone.0298601.s001.zip › Data/地级市Z_F工作报告.zip!/░▓╗╒/║╧╖╩╩╨/└┤╫╘╣1⁄2╓┌║┼í╛┬φ┐╦╩2╛▌═°í┐_2018.docx]

# **2018年政府工作报告**

发布时间：2018-01-15 08:12

【字体：[大](http://www.hefei.gov.cn/public/1741/javascript:void(0);) [中](http://www.hefei.gov.cn/public/1741/javascript:void(0);) [小](http://www.hefei.gov.cn/public/1741/javascript:void(0);)】

各位代表：

　　现在，我代表市人民政府向大会报告工作，请予审议，并请政协委员和其他列席人员提出意见。

****一、2017年和过去五年工作回顾****

　　2017年，在省委省政府和市委的坚强领导下，我们全面贯彻落实党的十八大、十九大和省市党代会精神，深入学习习近平新时代中国特色社会主义思想，以新理念引领新发展，坚持稳中求进工作总基调，大力实施五大发展行动计划，主动作为，砥砺奋进，在建设长三角世界级城市群副中心、“大湖名城、创新高地”的征程上阔步前行。

　　一年来，我们当好排头，经济实力再上台阶，主要指标稳居全国省会城市前十，对全省经济增长的贡献率进一步提升；我们勇担使命，科技创新再添动力，综合性国家科学中心、“中国制造2025”试点示范城市建设高位推进，量子科技创新研究院、江淮大众新能源汽车、引江济淮三个全省“一号工程”全面开工；我们敢破难题，改革开放激发活力，全面创新改革试验纵深推进，内陆开放新高地加快崛起；我们争创一流，城市建设管理提质提效，全国文明城市成功蝉联，城市功能进一步完善；我们善解民忧，全心全意办好民生实事，群众满意度、获得感进一步提升。经过全市上下共同努力，顺利完成了市十五届人大六次会议确定的主要目标任务。

　　一年来，我们主要做了以下工作：

　　（一）经济发展持续稳中向好。以供给侧结构性改革为主线，综合施策，精准调度，出台促进经济平稳健康发展“20条”和降低实体经济企业成本“10条”，开展“四送一服”，强化“四督四保”，经济发展保持较快增长，质量效益持续提升。预计，生产总值突破7000亿元、增长8.8%左右；规模以上工业增加值增长9%以上；固定资产投资6350亿元、增长5%；财政收入1251.1亿元、增长12.3%，其中地方财政收入655.9亿元、增长6.7%；社会消费品零售总额2730亿元、增长11.8%；进出口总额238亿美元、增长27%；居民人均可支配收入31800元、增长9.2%。节能减排指标全部完成省控目标。

　　（二）创新驱动实现重大突破。加快建设综合性国家科学中心、合芜蚌自主创新示范区，编制滨湖科学城、大科学装置集中区等重大规划，世界首条量子保密通信“京沪干线”正式贯通，聚变堆主机关键系统成功落户，科大讯飞智能语音入列国家人工智能四大平台，微尺度物质科学国家研究中心获批组建，离子医学中心、安徽创新馆等建设积极推进。深化与高校院所战略合作，推动科技创新与产业创新深度融合，中科大先研院、清华公共安全研究院、合肥北航科学城等创新平台建设加快推进，新建工程（技术）研究中心、企业技术中心、重点（工程）实验室258家，新增国家“双创”基地2家。出台人才新政“20条”，获批国家海外人才离岸创新创业基地，引进国家千人计划等高层次人才300余人，“哈佛八剑客”追梦科学岛成为人才回归、创新报国的时代佳话。

　　（三）产业转型迈出重大步伐。以“三重一创”建设为统领，新型显示、机器人国家区域集聚发展试点加快建设，6个省级战略性新兴产业集聚发展基地领跑全省，首批4个市级基地认定挂牌。高新技术产业增加值突破1400亿元，战略性新兴产业对工业增长的贡献率达到50%。全球首条最高世代线京东方10.5代液晶显示、康宁玻璃基板等项目竣工投产，站上了显示产业全球制高点。晶合12吋晶圆一期成功量产，在驱动芯片领域提升了国产化率，实现了“合肥芯”“合肥造”。家电“四大件”产量突破7000万台（套），联宝电子主营收入首超500亿元。完成技改投资1370亿元、增长8.5%，建成智能工厂41个、数字化车间320个。现代服务业加速发展，合肥商贸物流园区跻身全国首批示范园区，成功举办中博会、家博会等重大展会192场。

　　（四）改革开放取得显著成效。持续深化“放管服”改革，大力实施“多证合一”，新登记市场主体16.3万户。积极推进承担行政职能事业单位改革试点，建立事业编制周转池制度，组建数据资源局。调整市区财政分配体制，优化财力资源配置。出台深化国资国企改革实施意见，着力解决“三供一业”等历史遗留问题。健全企业上市融资服务机制，新增上市公司7家，直接融资3463.8亿元、增长47.9%。民营安徽新安银行正式开业。实施医药卫生体制综合改革，扎实推进医保管理体制“六统一”试点。稳步开展农村“三变”改革，全面完成土地确权颁证工作。出台打造内陆开放新高地实施意见，聚焦重点领域开展精准招商，引进大项目90多个。“合新欧”国际货运班列加密运行，合肥港吞吐量突破24万标箱，国际快件监管中心、航空港食用水生动物指定口岸通过验收，空港保税物流中心（B型）全面建成。设立“留学合肥”奖学金，有序推进中德教育合作示范基地等建设，50余家企业入驻“侨梦苑”。

　　（五）城市建设谱写崭新篇章。开展新一轮城市总体规划修编前期工作，推进全国首批城市设计试点市建设。新建续建大建设工程1514项，完成投资470亿元，六项工程荣获“鲁班奖”。合肥铁路枢纽规划获批实施。庐铜铁路全面建成，商合杭高铁等加快建设。滁淮高速、合宁扩容等工程进展顺利，合铜路二期等国省干线公路快速推进。轨道交通1号线运行平稳，2号线正式通车，单日最高客流量超过57万人次，3、4、5号线全面开建。阜阳路高架延伸、长江西路快速化改造等加快建设，上海路、绕城高速改线等全线贯通。启动长江中路街区等改造，开工红星路等慢行系统62条，推进海绵城市建设。新增公交车辆800台，建成500kV肥北变电站，五大类公共场所实现无线网络免费覆盖。以“绣花”功夫提升城市管理水平，城市净化、序化、绿化、亮化、美化水平全面提升。

　　（六）城乡统筹发展强力推进。持续加大县域发展支持力度，安排各类转移支付资金144亿元。加快农业产业化发展，市级以上现代农业产业化联合体发展到96家，新增新型农业经营主体1550家。“粮安工程”全面实施，粮食产量315万吨，肉蛋奶及水产品总量超过100万吨，主要“菜篮子”产品保障能力居全国大中城市第8位。大力开展土地综合整治，连续19年实现“占补平衡”。美丽乡村建设全面推进，完成34个乡镇政府驻地建成区整治任务，建成省、市级中心村278个。农村环境综合整治“三大革命”深入推进，完成陈年垃圾清理任务，改造农村厕所2.6万户。建成农村道路畅通工程5100多公里，行政村光纤网络实现全覆盖。灾后水利水毁修复工程顺利完成，滁河干渠江水西调工程正式通水。扎实开展“重精准、补短板、促攻坚”专项整改行动，1.6万建档立卡贫困人口脱贫。

　　（七）生态建设取得重大进展。出台加快生态文明建设实施方案、建设绿色发展美丽巢湖“20条”等，启动编制巢湖综合治理总体规划，强力抓好中央和省环保督察问题整改。统筹推进巢湖综合治理工程，建成十五里河污水处理厂三期和清溪净水厂。完善“湖长制”“河长制”，设置各级河长4600多名，双桥河、十五里河等重污染河流水质明显改善。实施蓝天行动，扎实开展餐饮油烟、扬尘等十大专项整治，PM10、PM2.5浓度分别为79.9、56微克/立方米，是全省唯一实现“双下降”的城市。出台土壤污染防治实施方案，启动4个修复试点项目。建立重点生态区域“林长制”，全年植树造林3.45万亩、城镇绿化1534万平方米。

　　（八）人民生活全面改善提升。高质量完成“31+9”项民生工程，倾心办好20项为民办实事事项。城镇新增就业24万人，城镇登记失业率下降至2.86%。实施全民参保计划，城乡低保、五保标准进一步提高。毫不动摇实施“十条新政”，积极探索租赁住房试点，有效遏制房价过快上涨势头，实现房地产市场平稳健康发展。新开工各类保障性住房2.62万套，改造城中村、危旧房项目9个，整治老旧小区192个。制定学前教育提升计划，出台中小学规划建设管理导则，启动“美丽校园”创建活动，建成城区幼儿园47所、中小学25所，合肥职业技术学院新校区、黄麓师范学校改扩建等加快建设。成功创建全国未成年人思想道德建设工作先进城市。大型原创舞剧《立夏》全国巡演，举办上海国际艺术节合肥分会场等活动，建成城市阅读空间24个。国家区域健康医疗大数据中心顺利获批，全国首家智慧医院挂牌成立，市儿童医院综合楼等建成使用，市公共卫生管理中心、全民健身中心等加快建设。加大治安立体防控力度，刑事案件发案起数下降15.8%。信访维稳形势总体向好，信访积案化解率近100%。扎实开展“百日除患铸安”专项行动，全力创建国家食品安全示范城市。国防动员和后备力量建设取得新进展。民族宗教、人防民防、防震减灾、统计、地方志、科普、气象、档案、保密等工作取得新成绩，工会、共青团、妇联、红十字会、老年人、残疾人和关心下一代等工作实现新进步。

　　各位代表！

　　过去一年，我们深入贯彻全面从严治党要求，认真开展“两学一做”学习教育和“讲重作”专题教育，不断提升政府自身建设水平。自觉接受人大监督，严格执行人大及其常委会的决议决定，主动接受政协民主监督、社会监督和舆论监督，全年办理人大代表议案和建议194件、政协提案628件。出台推进法治政府建设实施意见，完善重大行政决策程序，依法行政考核连续8年保持全省第一。全面实施“五项清单”，大力推进“互联网+政务服务”。严格落实党风廉政建设责任制，持续强化行政监察和审计监督，坚决查处各类侵害群众利益的腐败问题，持之以恒整“四风”、强作风。

　　各位代表！

　　五年来，本届政府积极应对复杂多变的宏观环境，保持定力、科学研判，开拓创新、奋发有为，经济社会发展取得重大成就。这五年，我们始终把创新驱动作为战略支撑，下好“先手棋”，抢占制高点，塑造更多依靠创新驱动的引领性发展。我们始终把工业立市作为强基之本，瞄准中高端，构建新体系，推动“合肥制造”迈向“合肥智造”。我们始终把生态保护作为永恒底线，坚守绿水青山，强攻大湖治理，努力让合肥的天更蓝、水更清、空气更清新。我们始终把优化环境作为重要保障，全力营造“四最”环境，充分激发干事创业活力。我们始终把满足人民美好生活期待作为至高追求，一件事情接着一件事情办，一年接着一年干，以政府辛苦指数换取百姓幸福指数。

　　——这五年，经济发展质量更优，综合实力大幅提升。坚持质量第一、效益优先，更好地提高质量、做大增量、扩张总量。生产总值连跨三个千亿台阶，年均增长10.1%，总量前进五位，居全国省会城市第10位。规模以上工业增加值年均增长11.4%，总量升至第9位。固定资产投资年均增长14.4%，总量升至第6位。财政收入年均增长12.5%，地方财政收入升至第10位。进出口总额稳居第9位。五年争先进位、跨越赶超，合肥开启了加速崛起的新征程。

　　——这五年，创新转型升级更快，核心竞争力显著提升。坚持以创新培育发展动能，以转型增强经济韧劲。基础研究取得重大突破，悟空探秘、墨子传信、热核聚变、铁基超导等一批具有国际领先水平的科技成果相继问世。主要创新指标进入省会城市前七，全社会研发投入占生产总值比重升至3.15%。以战略性新兴产业为先导、先进制造业和现代服务业为支撑的现代产业体系加快构建，智能语音及人工智能、集成电路、光伏太阳能、新能源汽车等保持国内领先，家电、新型显示等全国生产基地优势地位持续巩固；服务业占生产总值比重由39.8%提升至45.3%。新业态加速涌现，新经济快速发展。五年创新引领、厚积薄发，合肥抒写了建设创新之都的新华章。

　　——这五年，城市功能品质更好，承载辐射力有效提升。尊重城市发展规律，以现代城市理念提升规划建设品质。大建设工程累计投入1900多亿元。全国性综合交通枢纽地位确立，“米”字型高铁网络基本形成，新桥机场通航国际，合肥港通江达海，“一环八线”高等级公路网不断完善。合肥都市圈扩容升级，“5+2”一体化发展格局基本建立，与省内其他城市联动发展的态势初步形成。“1331”城市空间发展战略深入实施，城镇化率超过73%。城市步入地铁时代，快速路网加快构建，主城区与各组团基本实现“一刻钟快速交通”。农村基础设施与城市加快对接，教育、文化、卫生等公共服务均等化稳步推进。高新区、经开区、新站高新区、合巢经开区主引擎作用显著增强，辐射带动效应明显提升。肥西、肥东、长丰进入全国百强县，巢湖、庐江跻身全国中小城市投资潜力百强县市，包河、蜀山、瑶海、庐阳成为全国综合实力百强区。五年内外兼修、提升能级，合肥塑造了现代城市的新风貌。

　　——这五年，生态环境保护更严，持续发展力明显提升。绿色发展、生态优先理念深入人心，制度体系更加严密，生态环境持续改善。巢湖国家生态文明先行示范区建设取得阶段性成果。大气污染治理成效明显，空气质量优良天数持续增加。成功创建国家森林城市，森林覆盖率、城区绿化覆盖率分别提高到27.4%、46%，巢湖、肥东、肥西跻身国家园林城市（县城）。五年强力治污、生态保护，合肥描绘了绿色发展的新画卷。

　　——这五年，改革开放力度更大，社会创造力持续提升。坚持先行先试，全面深化改革多点发力、纵深推进。65项改革纳入国家试点，国资国企、财政金融、农业农村、行政审批和社会治理等重点改革取得突破。马（合）钢冶炼生产线整体关停，4800多名职工全部妥善安置。动态完善“1+3+5”产业政策体系，在全国率先探索光伏扶贫模式，省市共建公共资源交易“合肥模式”、高铁南站综合管理领跑全国，金融改革创出“一通三改”新模式。八大开放平台建设取得重大进展，与180多个国家和地区实现经贸合作，43家境外世界500强企业在肥投资。深度融入长三角一体化，加强与长江中游城市群联动发展，合肥上海双城合作渐入佳境。对台经贸和文化交流不断深化。结对合作扎实开展，阜合等共建产业园区建设稳步推进。援疆、援藏工作取得新进展。五年改革攻坚、开放合作，合肥彰显了革故鼎新、海纳百川的新气魄。

　　——这五年，获得感幸福感更强，民生保障力全面提升。坚持以人为本、民生优先，加快基本公共服务均等化、优质化。民生工程投入449亿元，惠及760余万群众。城镇新增就业近105万人。社会保障提标扩面，城乡基本养老、基本医疗保险、居民大病保险、被征地农民保障实现全覆盖。优质教育资源供给持续扩大，新建改扩建幼儿园306所、新建中小学150所。安徽名人馆等一大批公益文化设施建成开放，《合肥市志》《合肥通史》出版发行，连续荣膺中国最爱阅读城市，“大湖之约”艺术名家大讲堂形成品牌。138位市民入选“中国好人”，居全国省会城市第2位。三河古镇成功创建5A级景区，成为全国旅游标准化示范市。编制完成健康合肥2030规划，覆盖城乡的基层医疗卫生服务体系基本建成。全民健身成为风尚，环巢湖四季赛事打响品牌。平安合肥建设成效显著，社会大局和谐稳定。五年初心不改、躬身为民，合肥集聚了更加丰满的新福祉。

　　各位代表！

　　五年的成就令人瞩目，这是省委省政府和市委坚强领导的结果，是市人大依法监督、市政协民主监督、社会各界大力支持的结果，是全市人民团结奋斗、顽强拼搏的结果。在此，我代表市人民政府，向辛勤奋战在各个领域、各个岗位为合肥发展作出贡献的全市人民，向驻肥人民解放军、武警官兵、公安干警和中央驻肥单位，向各民主党派、工商联、无党派人士、各人民团体和社会各界人士，向所有关心支持合肥改革开放与现代化建设的海内外朋友，表示衷心的感谢和崇高的敬意！

　　在肯定成绩的同时，我们也清醒看到，当前合肥发展不平衡不充分的问题依然突出，主要是：经济发展质量和效益还不够高，传统优势产业亟需转型升级，战略性新兴产业仍需提速扩量，现代服务业发展相对滞后；城区转型不快，县域经济发展动能不足，区域协调发展任务繁重；巢湖综合治理、大气污染防治等任重道远；开放型经济发展水平不高，国际化都市区建设不快；大城市管理经验不足，精细化不够，交通拥堵等问题较为突出，教育、医疗、文化等基本公共服务均等化、优质化水平有待提升；效能建设仍需加强，少数政府部门和工作人员不作为、慢作为等现象仍有发生。对此，我们将高度重视，采取切实有效措施，认真加以解决。

****二、今后五年的奋斗目标和主要任务****

　　中国特色社会主义建设进入新时代，引领着合肥现代化建设走向更加美好的未来。新时代标注新方位。当前，全球新一轮科技革命和产业变革风起云涌，国际竞争合作方兴未艾，“一带一路”和长江经济带建设等纵深推进，为我们科学发展、跨越赶超提供了重大机遇和良好条件，合肥仍处于大有可为的战略机遇期。新时代承载新使命。进入发展新时代，社会主要矛盾已经转化为人民日益增长的美好生活需要和不平衡不充分发展之间的矛盾，经济已由高速增长阶段转向高质量发展阶段，我们要紧扣新矛盾、瞄准新目标，勇于担当，奋力争先，努力实现更高层次更高水平的新发展。新时代展现新作为。国家战略赋予合肥新的城市定位，省委省政府对合肥寄予厚望，全市人民对美好生活殷切期盼，我们要以永不懈怠的精神状态和一往无前的奋斗姿态，在决胜全面建成小康社会和社会主义现代化建设的新征程上再创佳绩。

　　今后五年，政府工作的总体要求是：高举中国特色社会主义伟大旗帜，以习近平新时代中国特色社会主义思想为指导，全面贯彻落实党的十九大精神，按照省市党代会和市委十一届五次全会精神，统筹推进“五位一体”总体布局和协调推进“四个全面”战略布局，坚持以新发展理念统领发展全局，坚持以满足人民日益增长的美好生活需要为目标，坚持以高质量发展为根本要求，坚定实施科教兴市、人才强市、创新驱动、乡村振兴、区域协调发展、可持续发展、军民融合发展等战略，大力实施五大发展行动计划，加快建设现代化经济体系，打造具有国际影响力的创新之都，在全国省会城市和长三角城市群中争先进位，在现代化五大发展美好安徽建设中作好表率、走在前列，奋力谱写社会主义现代化建设合肥篇章。

　　今后五年的主要奋斗目标是：发展质量稳步提高，创新动能显著增强，开放高地引领内陆，城乡融合更加协调，生态质量明显改善，文化建设繁荣兴盛，民生福祉全面提升，社会大局和谐稳定。全市生产总值超过1.1万亿元，人均生产总值突破2万美元，主要经济指标增幅和综合实力位居全国省会城市前列。

　　开启合肥现代化建设新征程，各县（市）区、开发区要找准新定位，实现新发展。四大开发区加快转型升级，推进产城融合，成为引领发展、辐射带动全市的高端产业集聚区、创新创业引领区、宜居宜业新城区。高新区全力建设世界一流高科技园区，经开区全力建设全球知名先进制造业集聚区，新站高新区全力建设世界级新型显示产业集群示范区，合巢经开区全力建设全国有影响的大健康产业集中区。瑶海、庐阳、蜀山、包河四个城区全力打造国际化都市区核心区、现代服务业集聚区、新经济新业态发展先行区、社会治理示范区。五县（市）全力增强新型工业化主战场作用，成为全市发展新引擎、全省县域发展排头兵，肥西、肥东、长丰巩固提升全国百强县位次，巢湖、庐江加快跻身全国百强县。

　　今后五年的主要任务是：

　　（一）融入全球创新体系，建设综合性国家科学中心。坚持把创新作为引领发展的第一动力，深入实施创新驱动发展战略，高标准规划国家级合肥滨湖新区，加快建设国际一流的滨湖科学城，全面提升参与全球科技竞争合作能力。加快建设具有国际影响力的大科学装置集群，高水平建成聚变堆主机关键系统等大科学装置，建设人工智能等交叉前沿研究平台，推动中科大先研院等创新平台形成示范效应。在量子信息、核聚变等领域形成一批具有国际领先水平的原创成果，在人工智能、网络安全、生命科学等领域形成一批引领性、颠覆性技术。以全球视野招才引智，引进培养集聚一大批国际水平的科技人才和高水平创新团队。系统推进全面创新改革试验，强化知识产权创造、应用和保护，构建最优创新生态圈。基本建成综合性国家科学中心，成为世界级科技创新策源地、国家级产业创新核心区、全国重要教育科研区。

　　（二）构建现代产业体系，建设中国制造强市。以供给侧结构性改革为主线，加快质量变革、效率变革、动力变革，系统构建现代产业体系，推动实体经济高质量发展。加快建设“中国制造2025”试点示范城市，推动新型显示、集成电路、光伏太阳能、智能语音及人工智能等战略性新兴产业发展成为世界级产业集群，促进汽车、家电、装备制造等优势产业升级成为全球制造研发基地，抢占量子信息、大数据、机器人、生命健康等未来产业发展先机。瞄准国际标准提高现代服务业发展水平，推动三次产业跨界和融合发展，高定位建设区域金融中心，把旅游业打造成重要支柱产业。以完善产权制度和要素市场化配置为重点，深入推进国资国企、商事制度、金融体制等改革。大力发展民营经济，培育一批“独角兽”和“隐形冠军”。扎实推进军民融合示范城市建设，加速壮大军民融合产业规模。

　　（三）提升城市功能品质，建设国际化都市区。贯彻“一尊重、五统筹”要求，加快建设以人为核心的现代化都市。建成全国性综合交通枢纽，构建现代交通体系，提升国际国内通达能力。完善“米”字型高铁网，衔接南京、福州、武汉、西安等9个方向，加快形成商合杭、合福、合宁、合武等13条线路引入的大型放射状枢纽。加密提标高速公路网，建成全国公路枢纽。深入推进智慧城市、信用城市建设，构建现代城市治理体系。持续实施城市畅通工程，构建轨道交通骨干网，建成快速路网体系和“公交都市”。全域优化功能布局，全面实施“多规合一”，开展城市设计试点，推进城市“双修”和“微更新”，高品质打造城市地标、特色街区和人文景观。

　　（四）全面扩大对外开放，建设内陆开放新高地。充分发挥“一带一路”和长江经济带双节点城市优势，积极融入全球开放体系，着力提升竞争合作水平。系统提升八大开放平台功能，加快建成全国性综合航空枢纽港、国家级临空经济示范区、全国一流的跨境贸易电商集聚区和产业示范区，全力打造江淮航运中心。构建与国际投资贸易通行规则相衔接的制度体系，积极争创以合肥为主片区的中国（安徽）自贸区。全面提升区域合作水平，拓展对德、对美、对俄等交流合作领域，深度融入长三角，联动长江中游城市群发展。扎实推进与皖北、皖西结对合作。增强核心作用，引领合肥都市圈一体化发展，打造安徽高质量发展的样板区。

　　（五）实施乡村振兴工程，建设融合发展先行区。坚持农业农村优先发展，建设产业兴旺、生态宜居、乡风文明、治理有效、生活富裕的美丽乡村。大力推进城乡融合发展，提档升级农村基础设施，逐步建立全面覆盖、普惠共享、城乡一体的基本公共服务体系。深化农业供给侧结构性改革，大力构建现代农业产业体系、生产体系、经营体系，加快提升产业园区发展水平，推进县域工业和都市农业增品种、提品质、创品牌。全面深化农业农村改革，巩固完善农村基本经营制度，保障农民财产权益。创新乡村治理体系，建设平安乡村。实施特色小镇培育计划，建成一批产业特色鲜明、生态环境优美、体制机制灵活、人文气息浓厚、宜业宜居宜游的特色小镇。

　　（六）聚力天蓝水清地绿，建设绿色生态美丽合肥。全面实施生态优先战略，构建人与自然和谐共生新格局。完善生态文明制度体系，严格实施生态环境监管。积极发展循环经济，加快建设低碳城市。大力建设绿色发展美丽巢湖，构建数字巢湖、全域监管、系统保护相结合的治理体系，基本建成引江济淮工程，流域水质明显好转，巢湖综合治理成为生态文明安徽样板。统筹山水林田湖草系统治理，严格保护耕地。坚持全民共治、源头防治，强化大气污染源监测预警和联防联控。积极创建国家生态园林城市，高品质建成森林生态网络，让绿化景观绘就城市最美生态画卷。

　　（七）树立高度文化自信，建设全国一流文化强市。坚定文化自信，持续提升城市文化品位和市民文化素养，建设一座有底蕴、有气质、有灵魂的城市。把中国梦和社会主义核心价值观融入到全市人民建设美好家园的生动实践中，汇聚成追求美好生活的强大精神力量。坚持以人民为中心的创作导向，推出更多反映时代、群众喜爱的精品力作，推动文创企业、文创园区等发展壮大，不断提升合肥文化对外影响力。打造全国一流的公共文化服务体系建设示范区，深入实施文化惠民工程，广泛开展群众性文化活动，努力建成全国乃至全球全民阅读典范城市。

　　（八）顺应人民美好生活期待，建设和谐宜居幸福之城。坚持民生至上，构建高质量公共服务体系，打造15分钟生活圈，努力实现幼有所育、学有所教、劳有所得、病有所医、老有所养、住有所居、弱有所扶。优先发展教育事业，打造教育强市，推动义务教育优质均衡发展，提升学前教育、特殊教育、网络教育水平，普及高中阶段教育，建设职教高地，支持“双一流”大学建设，创建合肥大学。实施就业优先战略和积极就业政策，提高就业质量，增加低收入者收入，扩大中等收入者比重。进一步完善统一的城乡居民基本医疗保险制度，稳步提高养老待遇水平，保障中低收入市民基本住房需求，完善城乡社会救助体系，积极探索新市民享有同城待遇的实现方式。大力实施精准扶贫和精准脱贫。建设健康合肥，深入开展全民健身运动，持续完善医疗卫生服务体系，推进医养结合，提高全方位全周期健康服务水平。深化平安合肥建设，推进社会治理现代化，构筑共建共治共享的社会治理新格局。

****三、2018年重点工作任务****

　　今年是全面落实党的十九大精神的第一年，是决胜全面建成小康社会、实施“十三五”规划承上启下的关键一年，是新一届政府的开局之年，我们要学习新思想，践行新理念，努力实现高质量发展。经济社会发展的预期目标是：全市生产总值增长8.5%左右；规模以上工业增加值增长9%左右；固定资产投资增长7%左右；财政收入增长10%左右；社会消费品零售总额增长10.5%左右；居民人均可支配收入增长快于经济增长；居民消费价格涨幅3%左右；城镇新增就业13万人，城镇调查失业率控制在5%以内；节能减排完成省控目标。

　　重点做好九个方面工作：

　　（一）着力推进经济发展质量变革。

　　提高供给体系质量。深化供给侧结构性改革，重点在“破”“立”“降”上下功夫。用市场化、法治化手段化解过剩产能，破除无效供给。积极创建全国质量强市示范城市，深化小微企业“双创”基地城市示范建设，培育共享经济、数字经济、人力资本服务等新业态新模式，加快壮大新动能。常态化推进“四送一服”双千工程，全面落实减税降费政策，切实降低企业成本。

　　扩大有效投入和消费需求。谋划储备一批基础设施、产业发展和民生工程类重大项目，高效推进300个亿元以上项目建设，建成180个以上。加大基础设施、社会事业和公用事业等领域开放力度，激发民间投资活力。推动互联网、大数据、人工智能和实体经济融合发展，促进大众消费升级。

　　提高金融服务实体经济能力。大力发展普惠金融，改善中小微企业金融服务，扩大政策性融资担保、“税融通”等新型融资规模，提升股权投资基金运作效率。加强后备资源培育，支持企业上市融资。打好防范化解重大风险攻坚战，全面加强地方金融监管和政府债务管控，开展非法违规金融活动打击、“互联网”金融专项整治、地方金融机构不良资产压降“三大行动”。

　　（二）着力推进创新发展动力变革。

　　建设综合性国家科学中心。按照国际标准规划建设滨湖科学城。高水平建设量子科技创新研究院，积极争创量子信息科学国家实验室、新能源国家实验室，加快聚变堆主机关键系统等大科学装置规划建设，基本建成离子医学中心，大力推进人工智能平台、天地一体化信息网络合肥中心等建设。

　　推进科技成果转移转化。推动中科大先研院等创新平台建立市场化运行机制，提升成果转化、企业孵化、产业培育能力。积极引进知名高校共建新型创新平台，推动中科大高新园区、合肥北航科学城、北外德国中心等建设取得重大进展。基本建成安徽创新馆。新增国家高新技术企业150户以上，新建工程研究中心、重点实验室等各类研发机构100家以上，有效发明专利拥有量、技术合同交易额增长15%以上。

　　优化创新生态环境。深化全面创新改革试验，出台科技成果转移转化专项政策，扩大科技成果“三权”改革等试点。落实人才新政“20条”，实施人才发展“6311”工程，建成合肥国际人才城。推进国家知识产权示范城市建设，新增示范企业100家以上。坚持“生态+创新”的理念，突出王咀湖、南艳湖、少荃湖等重点区域，以最美的自然生态集聚最高端的创新资源。

　　（三）着力加快产业转型升级。

　　加快发展先进制造业。积极建设“中国制造2025”试点示范城市，争创国家级示范区。统筹推进“三重一创”，以京东方、科大讯飞、晶合等为龙头，加快“中国声谷”等建设，集聚发展新一代信息技术产业，打造全国重要的人工智能产业先行区和智慧产业新高地；推动通威、晶澳等企业，巩固储能技术领先优势，加快阳光电源智慧能源管理系统建设，持续提升光伏太阳能产业核心竞争力；加快江淮大众、长安二期、大陆马牌轮胎四期等重大项目建设，推进海尔、格力、美的等家电企业智能化升级，促进产业链整体提升；积极培育量子通信等未来产业。推进智能制造“万千百”工程，新建智能工厂15个、数字化车间150个。建成军民融合信息平台，建立企业库、产品库和项目库，争创国家军民融合示范城市。

　　加快发展现代服务业。大力发展会展、物流、中介咨询等生产性服务业，培育发展健康养老、旅游休闲、家政服务等生活性服务业，提速发展工业设计、大数据、服务外包等高技术服务业，改造提升城区商圈服务功能。积极引进国际知名服务品牌和服务企业，聚力提升瑶海物联网产业园、庐阳金融集聚区、蜀山跨境电商园、包河创意文化战新基地、滨湖金融后台服务基地等发展水平。全力创建环巢湖国家旅游休闲区，推动巢湖市、庐江县创建国家全域旅游示范区。

　　扶持发展民营经济。放宽市场准入，加大政策支持，破除制约障碍，构建亲清新型政商关系，培育更多、更大、更强的民营企业。继续实施“专精特新”中小企业培育工程，培育一批专注细分领域的“单项冠军”和“配套专家”。积极倡导企业家精神、工匠精神，大力培养创新型企业家。

　　（四）着力提升城市功能品质。

　　优化城市空间布局。修编新一轮城市总体规划，编制完成全市空间规划等重大规划，提升县城规划，完善镇村规划。强化全域规划管控，推动东部新中心、骆岗机场、空港示范区等重点片区城市设计实施，构建中心城区、副中心、产业新城、小城镇、美丽乡村协调发展的新型城镇化格局。

　　建设综合交通枢纽。加快建设商合杭高铁、合安客专，开工建设合新高铁，完成合宁高铁前期，规划建设合肥新西站，建成高铁南站南广场。积极推进新桥国际机场二期，规划建设通用机场。全面建设引江济淮主体工程，开工建设派河港，推进合裕线航道船闸改造。加快实施合宁高速扩容等工程，开工建设合六高速扩容、明巢高速等工程，积极谋划外绕城高速连接线等前期工作。

　　完善城市承载功能。加快建设轨道交通3、4、5号线和1号线三期工程，积极推进6号线和2、3、4号线延长线。加密主城区与各组团快速通道，全面建成铜陵路、阜阳路高架延伸和长江西路快速化改造等重点工程，开工建设金寨路快速化改造等骨干路网工程，继续推进“畅通二环”建设。加快建设黄山路等公交专用道，完善城市慢行系统，有序实施城市支路和小街巷改造等“微循环”工程。开工建设龙河口引水工程，建成七水厂二期、绕城天然气高压管线、220kV大学城变电站等重点项目。

　　提高城市管理水平。巩固城市管理提升年成果，全面开展“两治三改”专项行动。实施城市出入口环境美化工程、骨干路桥亮化工程，推进长江中路等精品化改造，完成城隍庙片区等环境综合整治。加强重点时段和区域交通管理，加大公共停车场规划建设力度，鼓励市民绿色出行。建设新型智慧城市，构建城市智能运营平台，创建国家社会信用体系建设示范城市。

　　（五）着力推进乡村振兴。

　　推进县域经济振兴。大力实施制造强县战略，聚力发展主导产业，培育壮大特色产业，提升县域经济发展质量和层次。推进县域工业园区整合和优化升级，支持肥西桃花工业园、长丰双凤经济开发区争创国家级开发区，支持庐江县争创农业类国家级高新技术开发区。

　　提升都市现代农业水平。实施粮食产业三年提升行动，稳定粮食产能，强化“菜篮子”供给保障。推进农业多功能开发和标准化建设，做大做强高效绿色种养业，加快发展休闲农业和乡村旅游业、农产品加工流通业、定制农业、农产品电子商务等新兴业态，打造更多农产品知名品牌。提升农业产业化水平，大力建设现代农业示范区、现代农业园区，新增新型农业经营主体1000家以上。

　　大力改善农村设施环境。探索促进资本、技术、人才等要素向农业农村流动的政策措施。加强城乡规划和建设标准衔接，建成农村道路畅通工程3200公里，全面实施城乡客运公交化改造。巩固“百河千渠万塘”行动成果，提升农田水利设施建设水平。大力实施土地综合整治，新增耕地4万亩以上。深入推进美丽乡村建设，全面完成29个乡镇政府驻地建成区整治和70个中心村建设任务，启动建设80个中心村。深入开展农村环境“三大革命”，新建乡镇污水处理厂20个、中心村污水处理设施70个，完成农村改厕10万户以上。建立自治、法治、德治治理体系，推进乡村由“管理民主”向“治理有效”升级。

　　深入推进脱贫攻坚。深入实施脱贫攻坚十大工程，强化支撑保障体系，健全稳定脱贫长效机制，把扶贫和扶志、扶智结合起来，激发贫困人口内生动力，确保脱真贫、真脱贫，全面完成脱贫攻坚年度任务。

　　（六）着力深化改革攻坚。

　　深化经济领域改革。加快国有企业改制、整合和上市步伐，完善以“管资本”为主的国有资产分类监管体制。推进财政事权和支出责任划分改革，强化预算绩效管理。有序放开竞争性领域商品和服务价格，保持价格总水平基本稳定。深化统计管理体制改革。推进农村集体产权制度改革，稳步开展“三变”改革试点，加强农村承包地确权登记成果应用。

　　深化行政领域改革。持续深化“放管服”改革，全面推行权责清单制度，全方位加强事中事后监管。深层次推进商事制度改革，进一步加大“多证合一”整合力度。全面落实城市管理执法体制改革实施意见，理顺城市综合管理职责范围，推进执法标准化建设。持续推动公共资源交易管理改革创新，加快省市县一体化平台建设。积极推进投融资改革，稳步扩大PPP模式运用。

　　深化社会领域改革。加快社区治理体制改革，积极开展城乡社区协商和村（居）民自治试点。培育壮大社会组织，探索实践“五社联动”基层社会治理机制。完成机关事业单位养老保险制度改革。深入推进“三医联动”，全面完成医保管理体制改革试点任务。持续深化户籍制度改革，切实保障流动人口权益。

　　（七）着力提升开放水平。

　　增强开放平台功能。高标准建设国际内陆港，力争“合新欧”班列全年开行100列。推动合肥港发展多式联运，稳定提升港口吞吐量。开通航空港国际货运新航线，力争进境水果、冰鲜水产品、食用水生动物指定口岸成为区域分拣中心。拓展提升海关特殊监管区功能，高水平建设综合保税区、出口加工区。

　　大力开展招商引智。聚焦变革性技术研发、战略性新兴产业和先进制造业高端环节，全力引进领军人才、顶尖研发团队、知名研发机构，全链条引进重大产业项目。发挥“合肥之友”等平台作用，开展精准招商，加强与央企、知名民企、外企合作，办好2018世界制造业大会，全年招商引资4100亿元，其中外商直接投资32亿美元以上。

　　提高对外贸易水平。深入实施“走出去”战略，积极扩大高技术、高附加值、高效益产品出口，鼓励外贸企业在“一带一路”沿线等国家和地区建立营销服务网络。大力推动汽车、家电、装备制造、电子信息等优势产业走出国门，开展国际产能合作。

　　扩大对外交流合作。积极搭建合作平台，加快中德教育合作示范基地、中德（合肥）智慧产业园、智能制造创新园、“侨梦苑”等重点项目建设。加强国际友城等合作交流，争取举办高层次国际会议、高水平国际赛事。全面深化区域合作交流，深入推进上海合肥双城合作。推动合肥都市圈一体化发展，加强与省内城市联动发展。

　　（八）着力强化生态环境治理。

　　深化巢湖综合治理。高水平编制巢湖综合治理绿色发展总体规划。全面排查污染源，大力整治入河（湖）排水口。高标准实施巢湖综合治理三期、四期工程，开工建设五期工程，加快推进六期工程前期。推进胡大郢等污水处理厂建设，实施十五里河一期、望塘等污水处理厂提标改造。严格落实“湖长制”“河长制”，系统推进水资源保护、水污染防治、水生态修复和水环境治理。巩固黑臭水体治理成效，建立监管养护长效机制。

　　整治突出环境问题。深入开展亲水行动，加强董铺、大房郢水库等饮用水源和良好水体保护，集中攻坚南淝河、十五里河、派河等重污染河流治理，确保水质按期达标。打好蓝天保卫战，开展餐饮油烟污染专项整治，实行城区烟花爆竹禁放，严控道路、施工等扬尘，巩固秸秆禁烧成效。扎实推进净土行动，推动马（合）钢等土壤治理修复试点取得实效。严格工业固废、生活垃圾、医疗废物处置和监管。

　　提升城市绿化水平。全力创建国家生态园林城市，优化提升大蜀山、滨湖国家森林公园，高品质建设环巢湖绿色景观生态廊道，加快改造城市道路、广场和居民小区等绿化景观，新增公园游园、街头绿地40个以上。持续抓好森林长廊建设，完成植树造林5万亩，新增绿色长廊200公里、绿化面积600万平方米。推进庐阳三十岗等新十大城市公园建设，实施4个国家级湿地公园建设试点，积极申报国际湿地城市、申办中国（合肥）国际园林博览会。

　　严格生态环境监管。严守生态保护红线，划定永久基本农田和城市开发边界。强化排污者责任，健全环保信用评价、信息强制性披露、严惩重罚制度。严格落实“党政同责”“一岗双责”，严格执行考核评价和责任追究等制度，积极开展领导干部自然资源资产离任审计。健全网格化环境监管体系，加强区域联防联治，严厉打击环境违法违规行为。

　　（九）着力保障和改善民生。

　　繁荣发展文化事业。深入实施公民道德建设工程，深化群众性精神文明创建。大力实施全民科学素质行动计划，弘扬科学精神，普及科学知识。完善公共文化服务功能，积极推进市工人文化宫、中心图书馆建设，加快启动市科技馆新馆、美术馆、博物馆、妇儿活动中心、青少年活动中心、档案馆、方志馆等公益性项目，建成城市阅读空间50个。广泛开展群众喜闻乐见的文化活动，认真办好第十届中国曲艺牡丹奖大赛等全国性品牌文化活动。大力繁荣文艺创作，努力推出一批原创精品力作。

　　优先发展教育事业。办好学前教育，建成城区幼儿园47个，全面实现“明厨亮灶”。加快义务教育优质均衡发展，深入推进“三大提升工程”，实施特殊教育二期提升计划。深入创建“美丽校园”，新建改扩建中小学24个，建成合肥新四中、巢湖二中新校区，加快市青少年综合实践基地、市特教中心北校区建设。加大农村教育投入，缩小城乡教育发展差距。深入推进产教融合与校企合作，推动合肥职业技术学院、合肥幼儿师专建设地方技能型高水平大学；实施中等职业学校质量提升工程，完成黄麓师范学校改扩建等项目建设。继续推动合肥学院地方应用型高水平大学建设。强化师德师风建设，完善考核评价机制，提升教师队伍整体素质。

　　推进更高质量就业。鼓励创业带动就业，全面实施高校毕业生就业创业促进计划，不断提升四级就业创业公共服务网络功能。大规模开展就业培训，拓宽就业渠道。完善政府、工会、企业共同参与的协商协调机制，构建和谐劳动关系。

　　健全社会保障体系。建立统一的社会保险公共服务平台，完善各项社会保险关系制度衔接与跨区域转移接续政策。实施全民参保计划，提高城乡居民基础养老金和失业保险金标准，实现城乡养老保险体系全覆盖。统一城乡居民基本医保政策，全面实施按病种收付费结算。完善困难家庭救助机制，稳步提高城乡居民低保。从严落实房地产调控政策，稳步开展住房租赁试点，实施集体建设用地租赁住房试点项目，建立多主体供应、多渠道保障、租购并举的住房制度，筹集租赁住房1.5万套。启动11个城中村和危旧小区搬迁改造，新建棚户区改造安置房2万套，完成172个老旧小区综合整治任务。

　　加快健康合肥建设。大力发展大健康产业，推进国家区域健康医疗大数据中心建设，建成全民健康信息平台，加快建设智慧医院。推广家庭医生签约服务，推进分级诊疗，建设一批医养结合示范基地、示范社区。鼓励和规范社会力量办医，发展特色专科和高端医疗服务。加强应急队伍和装备建设，提升疾病预防控制和突发公共卫生事件应急处置能力。办好老百姓家门口的健身工程，打造10分钟健身圈，扩大合肥国际马拉松赛等品牌赛事影响力。加快城区农贸市场升级改造，扎实创建国家食品安全示范城市，保障人民群众“舌尖上的安全”。

　　加强社会治理创新。深入开展“七五”普法，全面推进法治合肥建设。加强预防和化解社会矛盾机制建设，加大领导干部接访下访力度，引导各类信访问题在法治轨道妥善解决。推进“智慧社区”建设，促进物业小区精细化管理。建立网络综合治理体系，营造清朗的网络空间。建设“雪亮工程”示范城市，打造立体化、信息化的社会治安防控体系。严格落实安全生产责任制，坚决遏制重特大安全事故。坚决响应改革强军号令，大力支持备战训练，全力推进双拥工作。进一步完善应急管理体系，全面提升综合防灾救灾能力。支持工会、共青团、妇联、红十字会等人民团体广泛参与社会管理和公共服务，扎实做好民族宗教、防震减灾、地方志、气象、新闻、档案、保密、残疾人等工作。

****四、加强政府自身建设****

　　新时代对政府自身建设提出了新任务、新要求，我们要以新担当、新作为，不断提高政府工作现代化水平。

　　以新思想引领方向。坚持用习近平新时代中国特色社会主义思想武装头脑，牢固树立“四个意识”，坚决维护党中央权威和集中统一领导，坚定维护习近平总书记党的领袖和核心地位。深入推进“两学一做”学习教育常态化制度化，巩固提升“讲重作”学习成果，组织开展“不忘初心、牢记使命”主题教育，奋力推动合肥各项事业新发展。

　　以新自觉依法行政。全面落实法治政府建设实施纲要，自觉运用法治思维和法治方式推动工作。依法接受市人大及其常委会工作监督和法律监督，自觉接受市政协民主监督，认真听取各民主党派、工商联、无党派人士和人民团体意见，认真办理人大代表议案建议和政协提案。完善行政执法和监督体系，推行重大行政决策民主公开。

　　以新标准执政为民。坚持以人民为中心，深入基层、深入一线，躬身倾听群众最深处的声音，努力做到“民有所呼，我有所应；民有所需，我有所为”。以人民群众满意作为评判政府工作的第一标准，抓住老百姓最急最忧最怨的问题，解决好群众最关心最直接最现实的利益问题，扎实开展为民办实事，让民生幸福的底色更厚重，让共享发展的主题更温暖。

　　以新定力廉洁从政。全面履行从严治党主体责任，严格遵守《廉洁自律准则》和《纪律处分条例》。健全完善惩治和预防腐败体系，强化审计监督。支持监察体制改革，实现对所有行使公权力的公职人员监察全覆盖。认真贯彻中央八项规定精神和省、市委实施细则，同形式主义、官僚主义的种种表现进行坚决斗争，锲而不舍纠正“四风”问题，坚决防止和纠正损害群众利益的违纪违法行为。

　　以新担当增强本领。以适应新时代的紧迫感，着力增强学习、政治领导、改革创新、科学发展、依法执政、群众工作、狠抓落实、驾驭风险的本领。坚持说实话、谋实事、出实招、求实效，勇于攻坚克难，以钉钉子精神做好各项工作。注重战略思维，自觉将合肥发展置于全球全国全省的大格局中来谋划，在敢闯敢试中探索新路、破解难题。

　　以新作风提升效能。持续推进简政放权，创优“四最”营商环境。大力推行“互联网+政务服务”，加快建成市县乡村一体化平台。完善政绩考核评价体系，健全正向激励机制，为敢于担当、勇于创新者撑腰鼓劲，严厉整治庸政懒政怠政，全面提升政府执行力和公信力。以永远在路上的坚持，抓实抓牢作风建设，真诚服务好科学家、企业家、千万家，使合肥真正成为养人的地方、创新的天地。

　　各位代表！

　　幸福都是奋斗出来的。让我们更加紧密团结在以习近平同志为核心的党中央周围，在省委省政府和市委的坚强领导下，高举中国特色社会主义伟大旗帜，凝心聚力，开拓创新，奋力谱写社会主义现代化建设合肥篇章，为决胜全面建成小康社会，实现中华民族伟大复兴中国梦作出新的更大贡献！
